# Neuroprotective activity of *Ulmus pumila* L. in Alzheimer's disease in rats; role of neurotrophic factors

**DOI:** 10.1016/j.heliyon.2020.e05678

**Published:** 2020-12-14

**Authors:** Rehab A. Hussein, Ahmed H. Afifi, Ahmed A.F. Soliman, Zeinab A. El Shahid, Khairy M.A. Zoheir, Khaled M. Mahmoud

**Affiliations:** a Pharmacognosy Department, Pharmaceutical and Drug Industries Research Division, National Research Centre, PO 12622, 33 El Bohouth St. (Former El Tahrir St.), Dokki, Giza, Egypt; b Chemistry of Natural and Microbial Products Department, Pharmaceutical and Drug Industries Research Division, National Research Centre, PO 12622, 33 El Bohouth St. (Former El Tahrir St.), Dokki, Giza, Egypt; c Cell Biology Department, Genetic Engineering and Biotechnology Research Division, National Research Centre, PO 12622, 33 El Bohouth St. (Former El Tahrir St.), Dokki, Giza, Egypt

**Keywords:** *Ulmus pumila L.*, Neuroprotective activity, Alzheimer's disease, Acetylcholinesterase inhibitors, BDNF, TGF-β1, Natural product chemistry, Public health, Neurology, Pharmacology, Alternative medicine

## Abstract

Alzheimer's disease (AD) is one of the most prevalent neurodegenerative disorders which affects the hippocampus and cortical neurons leading to impairment of cognitive ability. Treatment of AD depends mainly on acetylcholinesterase inhibitors, however, a novel therapeutic approach is introduced based on the maintenance of neuronal viability and functionality exerted through neurotrophic factors. In the current study, *Ulmus pumila* L. leaves alcoholic extract was investigated for its neuroprotective activity in AlCl_3_-induced AD in rats. Rats were orally treated with AlCl_3_ (17 mg/kg) for 4 weeks followed by *U. pumila* extract (150 mg/kg b.wt.) for another 6 weeks. Treatment of neuro-intoxicated rats with *U. pumila* extract resulted in a significant regulation in neurotrophic factors; brain derived neurotrophic factor and transforming growth factor-β and pro-inflammatory cytokine; TNF. It also induced an elevation in serum levels of monoamine neurotransmitters; norepinephrine, dopamine and serotonin and a decline in brain acetlycholinesterase activity. *U. pumila* extract also showed potent antioxidant activity as indicated by the declined malondialdehyde and elevated reduced glutathione, catalase and super oxide dismutase levels in AD rats‘ brains. Histological improvement was detected in the cerebral cortex, the hippocampus and striatum of the treated rats*.* The phytochemical analysis of *U. pumila* extract revealed high contents of flavonoids and phenolics and the major compounds were isolated and chemically characterized. Additionally, *U. pumila* extract and the isolated compounds exerted a prominent activity in *in-vitro* acetylcholinesterase inhibition assay with kaempferol-3-O-β-glucoside being the most potent compound showing IC_50_ of 29.03 ± 0.0155 μM. A molecular docking study indicated high affinity of kaempferol-3-O-β-robinobioside on acetylcholine esterase binding site with estimated binding free energy of –8.26 kcal/mol.

## 1 Introduction

Alzheimer's disease (AD) is one of the most prevalent neurodegenerative disorders (Calsolaro et al., 2019). The histopathology of AD includes two major hallmarks namely amyloid plaques (Aβ) and neurofibrillary tangles consisting of highly phosphorylated protein tau (p-tau) (Deture and Dickson, 2019). Accumulating data showed that various species of Aβ and tau were detected in cholinergic neurons of the basal forebrain system early in the course of the disease thus, triggering neuronal degeneration. Accordingly, drugs affecting acetylcholine (ACh) levels, basically acetylcholinesterase inhibitors (AChE-Is) were considered for the use as therapeutic agents for AD (Dall'acqua, 2013).

From another prospective, the role of neurotrophic factors in regulating neuronal survival and functionality has been recently depicted. Normal neuronal conservation and persistence requires activation of key signaling pathways which are triggered by these factors. Brain derived neurotrophic factor (BDNF) is one member of the neurotrophin family which is formed by neurons and glia. It attaches particularly to tropomyosin receptor kinase B (trkB) located on neurons leading to their provision. It also participates in maintaining synaptic plasticity. Transforming growth factor-β1 (TGF-β1) is another neurotrophic factor involved in the signaling pathways that imparts significant protective role in neurons (Wyss-Coray, 2006).

Additionally, the oxidative stress was investigated for the contribution in early stage of Alzheimer's disease preceding cytopathology as well as in the aggravation of the disease, inducing and activating multiple cells signaling pathways that contribute to lesion formations. Therefore, antioxidant therapies have displayed general success in preclinical studies of AD treatments (Feng and Wang, 2012).

*Ulmus pumila* L. family Ulmaceae, the Siberian elm, is a tree native to Central Asia, northern China and India. It has been used traditionally for inflammatory conditions and gastric cancer (Zhou et al., 2017). *Ulmus pumila* L. leaves showed cytotoxicity against human breast cancer cell line indicating its chemotherapeutic potential through induction of apoptosis (Hussien et al., 2019) This study aims to inspect the therapeutic potential of the alcoholic extract of *Ulmus pumila* L. leaves and the isolated compounds in ameliorating Alzheimer's Disease.

## 2 Materials and methods

### 2.1 Phytochemical study

#### 2.1.1 General experimental procedures

^1^H-NMR and ^13^C-NMR spectra were recorded on Bruker DRX 600 MHz and Bruker Avance III 400 MHz (Bruker Daltonics, Billerica, MA). ESI-MS spectra were recorded by “Waters” 3100 “USA", TQ Detector (Acquity ultra performance LC), Mass lynx V 4.1. Chemical shifts are given in values (ppm) relative to trimethylsilane as an internal reference. Silica gel 60, particle size 0.063–0.2mm, 70–230 mesh (Merck, Darmstadt, Germany) was used for column chromatography. For thin layer chromatography (TLC) aluminum sheet silica gel 60 F254 pre-coated plates (Merck, Darmstadt, Germany) were used.

#### 2.1.2 Preparation of *Ulmus pumila* L. Extract

A voucher specimen of *Ulmus pumila* L. was authenticated by Dr. Trease Labib, Herbarium of Orman Botanical Garden, Giza, Egypt. A weight of 500 g of *Ulmus pumila* L. leaves was extracted by 90% methanol (Fisher Scientific) at room temperature. The extract was filtered and evaporated to dryness under reduced pressure at a temperature not exceeding 40 °C, then the dried extract was deposited at the Extract Bank of the In-Vitro Bioassay Laboratory in the National Research Centre, Giza, Egypt.

#### 2.1.3 Estimation of total phenolic and total flavonoid contents

The total phenolic content of the extract was determined using Folin-Ciocalteu method (Kujala et al., 2000) expressed as mg gallic acid equivalent (GAE)/g extract. Total flavonoid content was determined by Davis deformed method (Lamson and Brignall, 2000) and expressed as mg rutin equivalent (RE)/g extract.

#### 2.1.4 Separation and identification of the major compounds of *Ulmus pumila* L. Extract

*Ulmus pumila* L. extract (14.5 g) was subjected to liquid-liquid partitioning between water and ethyl acetate. The ethyl acetate fraction (4 g) was further partitioned between hexane and 90% aqueous methanol. The latter fraction was chromatographed over silica bed (silica gel 60) using mixture of methanol in dichloromethane as mobile phase starting from 0% to 100% methanol to afford ten fractions (A-J). Fraction B (150 mg) eluted with 5% MeOH/CH_2_Cl_2_ was chromatographed on sephadex LH-20 (Sigma) using 50% MeOH/CH_2_Cl_2_ as mobile phase to yield nine fractions of which fraction B-7 (50 mg) was further purified by reversed phase C-18 flash chromatography (Buchi-Reverelis Prep., Reverelis® phase C18 cartridge) with gradient elution from 0% to 100% MeOH in H_2_O to yield**1** (8 mg) and **2** (5.5 mg). Fraction C (100 mg) eluted with 10% MeOH/CH_2_Cl_2_ subjected to further purification on sephadex LH-20 with 50% MeOH/CH_2_Cl_2_ as mobile phase to yield **3** (10 mg) and **4** (3 mg). Fraction D (120 mg) eluted with 15% MeOH/CH_2_Cl_2_ yielded **5** (9.5 mg) and **6** (8 mg) after purification on sephadex LH-20. Finally, fraction E eluted with 20% was purified on sephadex LH-20 and eluted with 100%MeOH to afford **7** (4 mg) and **8** (2.5 mg).

### 2.2 Molecular docking study

3D structures of compounds isolated from the bioactive extract were built by mean of Molecular-Builder program creating a database which was used as input file in MOE-docking. The energies of compounds were minimized to 0.05 Gradient using MMFF94x force field. The receptor was protonated and its energy was minimized to 0.05 Gradient using Amber99 forcefield. The database was docked into the active site of the Acetylcholinesterase receptor using the Triangular Matching docking method and 30 conformations of each Ligand protein complex were generated with docking score (S). Each complex was analyzed for interactions and their 3D pose was taken (Rahim et al., 2015).

### 2.3 Pharmacological study

#### 2.3.1 *In-vitro* assays

##### 2.3.1.1 Antioxidant activity

Antioxidant activity of *U. pumila* extract was determined by DPPH radical scavenging assay (Mishra et al., 2012)Superoxide anion scavenging assay (Siddhuraju and Becker, 2007) and Reducing power assay (Ferreira et al., 2007).

##### 2.3.1.2 Acetyl cholinesterase (AChE) inhibitory activity

*U. pumila* extract was solubilized in methanol (HPLC). The reaction mixture contained 150 μL of (100 mM) sodium phosphate buffer (pH 8.0), 10 μL of 5,5-Dithiobis-(2-nitrobenzoic acid) (DTNB, Sigma), 10 μL of test-extract solution and 20 μL of acetyl cholinesterase solution (Electric-eel AChE- Sigma) were mixed and incubated for 15 min (37 °C) followed by the addition of 10ml of acetylthiocholine (Sigma) to initiate the reaction. Hydrolysis of acetylthiocholine was monitored by the formation of yellow 5-thio-2- nitrobenzoate anion measured by UV- Vis Shimadzu spectrophotometer-USA at a wavelength of 412 nm (15 min).

Statistical analysis: reactions were carried out in triplicates and the data were expressed as mean ± SEM. The inhibitory concentration (IC50) of the total extract and each compound, was graphically evaluated by a non-linear regression method using Graph Pad Prism (Ver. 5.0) software.

#### 2.3.2 Animal study

##### 2.3.2.1 Acute toxicity study

Twenty Swiss mice of 20–30 g body weight was used for acute toxicity assay. *Ulmus pumila* L. extract was suspended in distilled water and orally administered to mice in gradually increasing doses starting from 250 mg/kg mice body weight and up to 2000 mg/kg body weight. A control group receiving equivalent volumes of distilled water was used. The 24 h mortality counts among equal sized groups of lethally intoxicated mice (8 animals/group) were estimated and the LD50 was calculated. Observation of mice was kept for 14 days, for any changes in skin, hair, activity, food intake, water consumption and body weights (Ammar et al., 2013).

##### 2.3.2.2 AlCl_3_-induced neurotoxicity in rats

###### 2.3.2.2.1 Animals

Male Wistar rats (180–200 g) procured from Central Animal House, National Research Centre (NRC), were acclimatized to laboratory conditions at room temperature with food and water ad-libitum in plastic cages with soft bedding. The protocol was approved by the NRC Ethics Committee (approval no. 19039) in accordance with the European community guidelines for the use and care of animals.

###### 2.3.2.2.2 Chemicals

Aluminum chloride (AlCl_3_) was purchased from BDH Laboratory Supplies, Poole UK, Donepezil tartrate was purchased from Sigma, USA and TRIzol reagent was bought from Invitrogen (Germany). The reverse transcription (RT) and polymerase chain reaction (PCR) kits were obtained from Fermentas (USA). SYBR Green Mix was purchased from Stratagene (USA). All other chemicals used were purchased from standard commercial suppliers and were of analytical grade quality.

###### 2.3.2.2.3 Experimental design

Animals were randomized into four groups of 8 rats each distributed as follows: Group 1; normal healthy rats served as untreated negative control group, Group 2; positive control group where the rats were orally administered with AlCl_3_ (17 mg/kg, p.o.) (Zaher et al., 2020) daily throughout the whole experiment, Group 3; rats were treated with daily oral dose of *Ulmus pumila* L. extract (150 mg/kg b.wt) for six weeks after 4 weeks intoxication with AlCl_3_. Group 4: rats orally administered daily with standard drug (Donepezil tartrate; 10 mg/kg b.wt./day) (Kandiah et al., 2017) after 4 weeks intoxication with AlCl_3_.

###### 2.3.2.2.4 Behavioral study (Y maze)

The animals' behavioral activities, including spatial learning, age related cognitive decline and memory, were studied using the Y maze test (Foyet et al., 2015). The maze used in the present study consisted of three arms (35 cm long, 25 cm high and 10 cm wide). Each of the three arms can be sealed off with a door, limiting the space that the rodent has to access. During the test a rat from each group is placed in an arm and one of the remaining two arms is closed off. The open arm, however, contains a food reward. The rat will roam and find the food reward. Then, in the next round, the other arm is now sealed off. During testing, when both arms are open, a mouse is to alternate between arms in consecutive trials. So, a rat is placed at the starting position, and finds reward in one arm. Then, a new trial begins and a rat is returned to the start, then it is expected to go down the other arm. All animals were tested in a randomized order at the start and end of the experimental protocol. Thirty minutes after rats treatment with either *U. pumila* extract (150 mg/kg b.wt) or reference drug (Donepezil 10 mg/kg b.wt./day); rats were placed at the end of one arm and allowed to move freely through the maze for 8 min. The time limit in Y-maze test was 8 min, and every session was stopped after 8 min or when the rat reaches the food reward. The maze was wiped clean with 70 % ethanol between each animal to minimize odor cues.

###### 2.3.2.2.5 Blood and brain tissue sampling

By the end of the experiment, rats were fasted overnight, blood samples were collected from the sublingual vein. Blood samples were centrifuged at 4000 RPM for 10 min and sera were frozen at −20 °C for biochemical analysis.

Rats were then sacrificed by cervical decapitation. Brains were rapidly dissected, washed with isotonic saline, and dried on filter paper. Each brain was divided sagittally into two portions. The first portion was weighed and homogenized, using Omni thq - digital tissue homogenizer- USA, in ice-cold medium containing 50 mMTris/HCl and 300 mM sucrose at pH 7.4 to give a 10% (w/v) homogenate. The homogenate was centrifuged at 3000 rpm for 10 min at 4 °C. The supernatant was separated for biochemical analysis. The second portion of each brain was fixed in formalin buffer (10%) for histopathological investigation. The ethical conditions were applied such that the animals suffered no pain at any stage of the experiment, and the study was approved by the Ethics Committee of the NRC. Animals were disposed of in bags provided by the Committee of Safety and Environmental Health, NRC.

###### 2.3.2.2.6 Monoamine neurotransmitters estimation

Serum levels of dopamine, serotonin and norepinephrine were determined by enzyme linked immunoassay using BioTech- ELISA reader- USA according to (Gaballah et al., 2016).

###### 2.3.2.2.7 Acetylcholine esterase estimation

AChE activity was estimated in the whole brain homogenates according to Ellman's method (Khan et al., 2012). Briefly, the brain homogenate was incubated for 5 min with 2.7 ml of phosphate buffer and 0.1 ml of 5, 5-dithiobis (2-nitrobenzoate) (DTNB). Further, 0.1 ml of freshly prepared acetylcholine iodide (pH 8) was added, and the change in absorbance was recorded at 412 nm.

###### 2.3.2.2.8 Antioxidant parameters assessment

Assessment of Superoxide dismutase (SOD), catalase (CAT), lipid peroxidation and reduced glutathione (GSH) activities were estimated in serum and tissue homogenate according to Ahmed and Nabil (2007) (Ahmed and Nabil, 2007).

###### 2.3.2.2.9 Assessment of neurotrophic factors

RNA was isolated from the brain tissue homogenate using TRIzol reagent according to the manufacturer's instructions and quantified by measuring the absorbance at 260 nm. RNA quality was determined by measuring the 260/280 absorbance ratio. Quantitative analysis of mRNAs was performed by RT-PCR through subjecting the resultant cDNA to PCR amplification using 96-well optical reaction plates in the ABI Prism 7500 System (Applied Biosystems). The 25μl reaction mixture contained 0.1 μl of 10 μM forward primer and 0.1 μl of 10 μM reverse primer (40 μM final concentration of each primer), 12.5 μ1 of SYBR Green Universal Master mix, 11.05 μ1 of nuclease-free water, and 1.25 μ1 of cDNA sample. Primers used in the current study were chosen from pubmed.com (http://www.ncbi.nlm.nih.gov/tools/primer-blast) as listed below. Assay controls were incorporated onto the same plate, namely, no-template controls to test for the contamination of any assay reagents. The real-time PCR data have been analyzed using the relative gene expression (i.e., ÄÄCT) method, as described in Applied Biosystems, User Bulletin No. 2. Briefly, the data are presented as the fold change in gene expression normalized to the endogenous reference gene (GAPDH) and relative to a calibrator.

Tumor necrosis factor (Tnf), mRNA (NM_001278601.1)F: GCGGAGTCCGGGCAGGT CTA, R: GGGGGCTGGCTCTGTGAGGA. Transforming growth factor, beta 1 (Tgfb1), mRNA (NM_011577.2); F: GCTGAACCAAGGAGACGGAA, R: AGAAGTTGGCATGGTA GCCC. *Mus musculus* brain derived neurotrophic factor (Bdnf), transcript variant 12, mRNA (NM_001316310.1); F: AGGGTCTGCGGAACTCCAG, R: GTTTGTTCGGCTCCACTGAG and GAPDH; F: CCCAGCAAGGACACTGAGCAAG, R: GGGTCTGGGATGGAAATTGTG AGGG.

###### 2.3.2.2.10 Statistical analysis

Data are presented as mean ± SEM. Statistical analysis was carried out using one-way analysis of variance (ANOVA) followed by Tukey's multiple comparison test to judge the difference between the various groups. Statistical significance was acceptable to a level of *P<0.05*. Data analysis was accomplished using the software program GraPad Prism (version 5).

###### 2.3.2.2.11 Histopathological examination

Autopsy samples from the rat's brains were fixed in 10% formol saline for 24 h. Washing was done in tap water then serial dilutions of alcohol were used for dehydration. Specimens were cleared in xylene and embedded in paraffin at 56 °C in hot air oven for 24 h. Paraffin bees wax tissue blocks were prepared for sectioning at 4 microns thickness by sledge microtome. The obtained tissue sections were collected on glass slides, deparaffinized and stained by hematoxylin &eosin stain for examination through the light electric microscope (Downie, 1990).

## 3 Results

### 3.1 Phytochemical investigation of *Ulmus pumila* L. Extract

The results of the spectrophotometric estimation of total phenolic and flavonoid contents showed that *Ulmus pumila* L. extract possessed remarkably high amounts of phenols (175.9 ± 5.2mg) gallic acid equivalent/gram extract and total flavonoid content of 68.7 ± 1.2 mg rutin equivalent/g extract.

**Figure 1:**
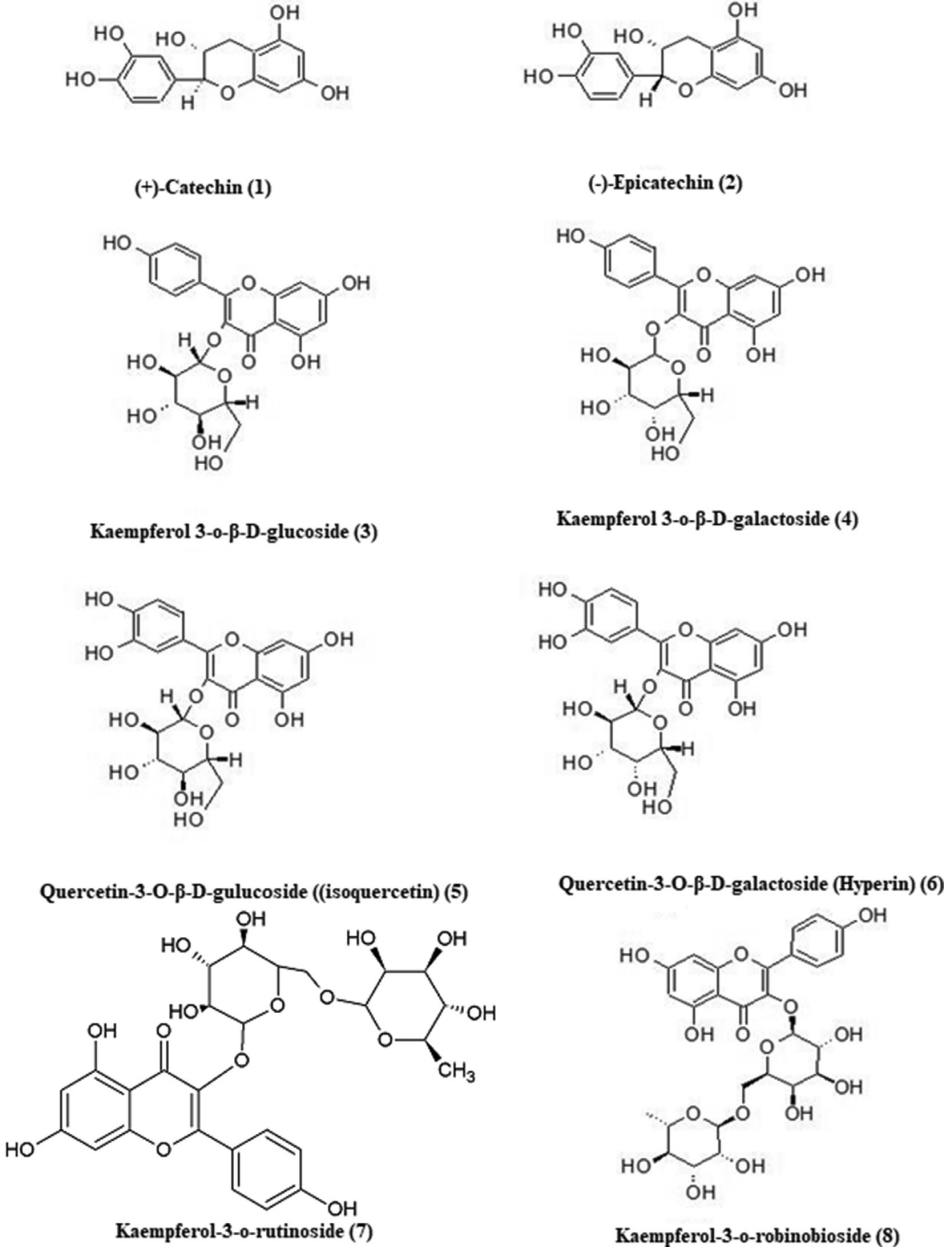
Chemical structures of compounds isolated from *U. pumila* extract.

Repeated chromatographic steps led to the separation and purification of eight flavonoids (Figure 1). The isolated flavonoids were identified as (+)-catechin **(1),** (-)-epicatechin **(2),** kaempferol 3-o-β-D-glucoside **(3),** kaempferol 3-o-β-D-galactoside**(4),** quercetin-3-O-β-D-gulucopyranoside ((isoquercetin) **(5),** quercetin-3-O-β-D-galactopyranoside (Hyperin) **(6),** kaempferol 3-o-rutinoside **(7),** Kaempferol 3-o-robinobioside **(8)** by comparison of their ^1^H- NMR and ^13^C-NMR with those reported in the literature (Lee et al., 2012; Panda and Kar, 2007; Brasseur and Angenot, 1986; Leong et al., 2008; Masika et al., 2004; Agrawal, 1989).

**(+)-Catechin (1):** ESI-MS m/z: 291 [M + H]. ^1^H-NMR (CD3OD, 600 MHz): δ 6.84 (1H, d, *J* = 1.8 Hz, H-2′), 6.76 (1H, d, *J* = 8.1 Hz, H-5′), 6.72 (1H, dd, *J* = 1.8, 8.1Hz, H-6′), 5.93 (1H, d, *J* = 2.3 Hz, H-6), 5.86 (1H, d, *J* = 2.3 Hz, H-8), 4.56 (1H, d, *J* = 7.5 Hz, H-2), 3.98(1H, m, H-3), 2.85 (1H, dd, *J* = 5.4, 16.1 Hz, H-4α), and 2.51 (1H, dd, J = 8.1, 16.1 Hz, H-2β). ^13^C NMR (150 MHz, CD3OD) δ 82.85 (C-2), 68.80 (C-3), 28.51 (C-4), 157.57 (C-5), 96.30 (C-6), 157.83 (C-7), 95.51 (C-8), 156.91 (C-9), 100.83 (C-10), 132.21 (C-1′), 115.26 (C-2′), 146.23 (C-3′), 146.24 (C-4′), 116.09 (C-5′), 120.04 (C-6′).

**(-)-Epicatechin (2):** ESI-MS m/z: 291 [M + H]. ^1^H-NMR CD3OD, 600 MHz): δ: 6.98 (1H, d, *J* = 1.9 Hz, H-2′), 6.80 (1H, dd, *J* = 1.9, 8.1Hz, H-6′), 6.76 (1H, d, *J* = 8.1 Hz, H-5′), 5.94 (1H, d, *J* = 2.3 Hz, H-6), 5.92 (1H, d, *J* = 2.3 Hz, H-8), 4.82 (1H, brs, H-2), 4.18(1H, brs, H-3), 2.74 (1H, dd, *J* = 2.9, 16.6 Hz, H-4α), and 2.86 (1H, dd, J = 4.6, 16.6 Hz, H-2β). ^13^C NMR (150 MHz, CD3OD) δ 79.87 (C-2), 67.48 (C-3), 29.25 (C-4), 157.67 (C-5), 96.22 (C-6), 158.00 (C-7), 95.89 (C-8), 157.37 (C-9), 100.08 (C-10), 132.29 (C-1′), 115.33 (C-2′), 145.94 (C-3′), 145.78 (C-4′), 115.90 (C-5′), 119.40 (C-6′).

**Kaempferol 3-o-β-D-glucoside (3):** ESI-MS m/z: 449 [M + H]. ^1^H-NMR (DMSO-d6, 400 MHz): δ: 12.62 (br. s, 1H, C5–OH), 8.04 (2H, d, *J* = 8.8 Hz, H-2′, 6′), 6.88 (2H, d, *J* = 8.8 Hz, H-3′, 5′), 6.42 (1H, d, *J* = 1.6 Hz, H-6), 6.20 (1H, d, *J* = 1.6 Hz, H-8), and 5.45 (1H, d, *J* = 7.2, glucose H-1). ^13^C NMR (150 MHz, DMSO-d6) δ 156.31 (C-2), 133.24 (C-3), 177.51 (C-4), 161.28 (C-5), 98.82 (C-6), 164.42 (C-7), 93.77 (C-8), 156.48 (C-9), 104.00 (C-10), 120.97 (C-1′), 130.96 (C-2′, 6′), 115.19 (C-3′, 5′), 160.02 (C-4′), 100.93 (C-1″), 74.28 (C-2″), 77.54 (C-3″), 69.96 (C-4″), 76.48 (C-5″), 60.90 (C-6″).

**Kaempferol 3-o-β-D-galactoside (4):** ESI-MS m/z: 449 [M + H]. ^1^H-NMR (DMSO-d6, 400 MHz): δ: 12.62 (br. s, 1H, C5–OH), 8.07(2H, d, *J* = 8.8 Hz, H-2′, 6′), 6.86 (2H, d, *J* = 8.8 Hz, H-3′, 5′), 6.42 (1H, d, *J* = 1.6 Hz, H-6), 6.20 (1H, d, *J* = 1.6 Hz, H-8), and 5.40 (1H, d, *J* = 7.6, galactose H-1). ^13^C NMR (150 MHz, DMSO-d6) δ 156.41 (C-2), 133.30 (C-3), 177.58 (C-4), 161.28 (C-5), 98.82 (C-6), 164.42 (C-7), 93.77 (C-8), 156.48 (C-9), 103.95 (C-10), 120.94 (C-1′), 131.05 (C-2′, 6′), 115.15 (C-3′, 5′), 160.02 (C-4′), 101.76 (C-1″), 71.27 (C-2″), 73.17 (C-3″), 67.95 (C-4″), 75.83 (C-5″), 60.26 (C-6″).

**Quercetin-3-O-β-D-gulucopyranoside ((isoquercetin) (5):** ESI-MS m/z: 465 [M + H]. 1H-NMR (DMSO-d6, 400 MHz): δ: 12.**64** (br. s, 1H, C5–OH), 7.58 (1H, dd, *J* = 8.5, 2.0 Hz, H-6′), 7.57 (1H, d, *J* = 2.0 Hz, H-2′), 6.83 (1H, d, *J* = 8.5 Hz, H-5′), 6.40 (1H, d, *J* = 1.8Hz, H-8), 6.20 (1H, d, *J* = 1.8 Hz, H-6), and 5.46 (1H, d, *J* = 7.2, galactose H-1). ^13^C NMR (150 MHz, DMSO-d6) δ 156.50 (C-2), 133.70 (C-3), 177.6**3** (C-4), 161.34 (C-5), 98.82 (C-6), 164.24 (C-7), 93.63 (C-8), 156.53 (C-9), 10**4**.2**0** (C-10), 12**1**.42 (C-1′), 1**15.24** (C-2′), **144**.83 (C-3′), 148.53 (C-4′), 116.51 (C-5′), 121.64 (C-6′), 101.41 (C-1″), 74.27 (C-2″), 76.79 (C-3″), 70.24 (C-4″), 77.46 (C-5″), 61.33 (C-6″).

**Quercetin-3-O-β-D-galactopyranoside (Hyperin) (6):** ESI-MS m/z: 465 [M + H]. ^1^H-NMR (DMSO-d6, 400 MHz): δ: 12.**64** (br. s, 1H, C5–OH), 7.67 (1H, dd, *J* = 8.5, 2.0 Hz, H-6′), 7.53 (1H, d, *J* = 2.0 Hz, H-2′), 6.83 (1H, d, *J* = 8.5 Hz, H-5′), 6.40 (1H, d, *J* = 1.8Hz, H-8), 6.20 (1H, d, *J* = 1.8 Hz, H-6), and 5.37 (1H, d, *J* = 7.7, galactose H-1). ^13^C NMR (150 MHz, DMSO-d6) δ 156.**3**0 (C-2), 133.**8**0 (C-3), 177.5**3** (C-4), 161.24 (C-5), 98.**6**2 (C-6), 164.04 (C-7), 93.**44** (C-8), 156.**31** (C-9), 10**4.00** (C-10), 12**1.3**2 (C-1′), 1**15.24** (C-2′), **144.7**2 (C-3′), 148.53 (C-4′), 116.21 (C-5′), 121.83 (C-6′), 102.31 (C-1″), 71.27 (C-2″), 73.44 (C-3″), 68.04 (C-4″), 75.76 (C-5″), 60.83 (C-6″).

**Kaempferol-3-o-rutinoside (7):** ESI-MS m/z: 595 [M + H]. ^1^H-NMR (DMSO-d6, 400 MHz): δ: 12.57 (br. s, 1H, C5–OH), 7.98(2H, d, *J* = 8.8 Hz, H-2′, 6′), 6.88 (2H, d, *J* = 8.8 Hz, H-3′, 5′), 6.41 (1H, d, *J* = 1.9Hz, H-6), 6.20 (1H, d, *J* = 1.9 Hz, H-8), and 5.30 (1H, d, *J* = 7.2, glucose H-1), 4.37 (1H, brs, Rhamnose H-1), 0.98 (3H, d, *J* = 6.0, Rhamnose H-6). ^13^C NMR (150 MHz, DMSO-d6) δ 156.60 (C-2), 133.50 (C-3), 177.45 (C-4), 161.34 (C-5), 98.82 (C-6), 164.04 (C-7), 93.84 (C-8), 156. 92 (C-9), 104.21 (C-10), 121.42 (C-1′), 130.90 (C-2′, 6′), 115.22 (C-3′, 5′), 159.82 (C-4′), 101.53 (C-1″), 74.20 (C-2″), 76.51 (C-3″), 70.11 (C-4″), 75.80 (C-5″), 66.94 (C-6″), 100.62 (C-1‴), 70.30 (C-2‴), 70.74 (C-3‴), 72.02 (C-4‴), 68.13 (C-5‴), 17.43 (C-6‴).

**Kaempferol-3-o-robinobioside (8):** ESI-MS m/z: 595 [M + H]. ^1^H-NMR (DMSO-d6, 400 MHz): δ: 12.57 (br. s, 1H, C5–OH), 8.05(2H, d, *J* = 8.8 Hz, H-2′, 6′), 6.86 (2H, d, *J* = 8.8 Hz, H-3′, 5′), 6.42 (1H, d, *J* = 1.8Hz, H-6), 6.20 (1H, d, *J* = 1.8 Hz, H-8), and 5.30 (1H, d, *J* = 7.2, galactose H-1), 4.39 (1H, brs, Rhamnose H-1), 1.05 (3H, d, *J* = 6.0, Rhamnose H-6). ^13^C NMR (150 MHz, DMSO-d6) δ 156.50 (C-2), 133.30 (C-3), 177.55 (C-4), 161.24 (C-5), 98.82 (C-6), 164.04 (C-7), 93.77 (C-8), 156.49 (C-9), 103.81 (C-10), 120.92 (C-1′), 131.05 (C-2′, 6′), 115.12 (C-3′, 5′), 160.02 (C-4′), 102.11 (C-1″), 71.17 (C-2″), 73.14 (C-3″), 68.14 (C-4″), 73.72 (C-5″), 65.43 (C-6″), 100.12 (C-1‴), 70.73 (C-2‴), 70.41 (C-3‴), 72.02 (C-4‴), 68.31 (C-5‴), 17.91 (C-6‴).

### 3.2 Molecular docking study

**Figure 2:**
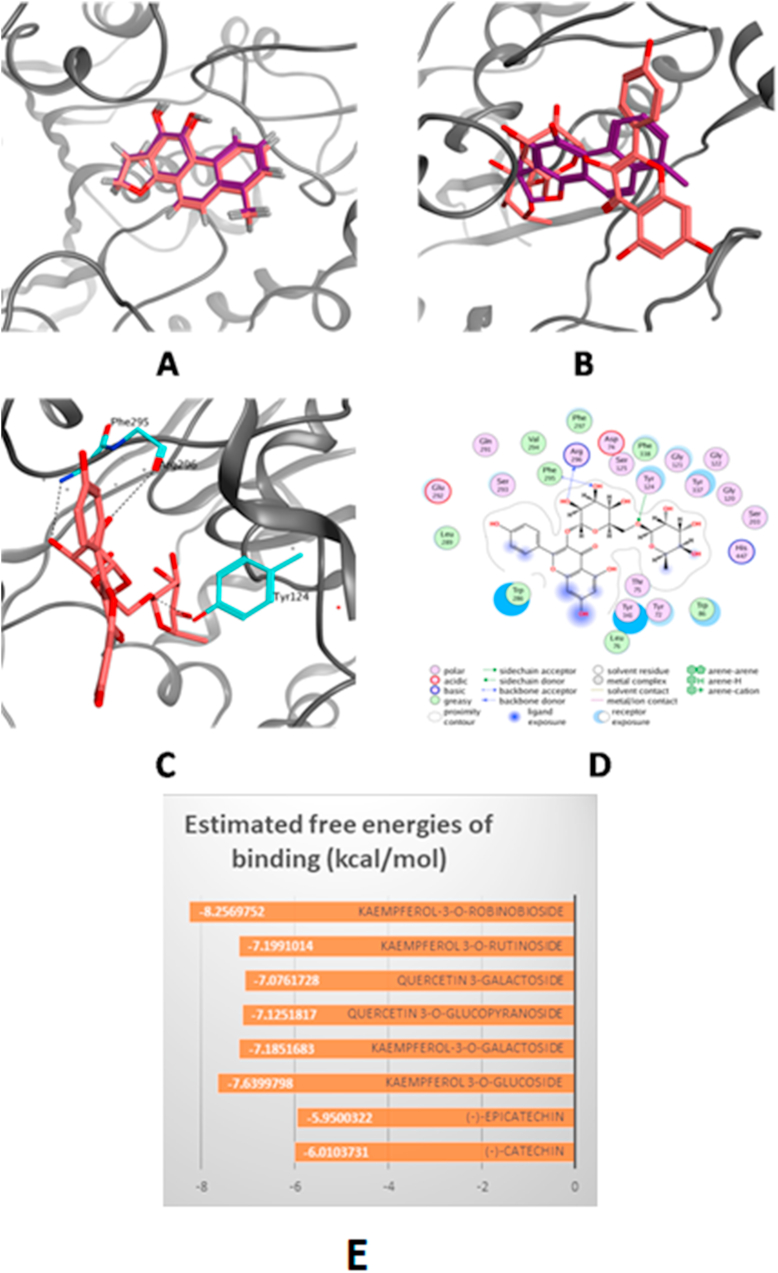
The top-scoring docked pose of Kaempferol 3-o-robinobioside to the AChE active site (PDB code 4M0E) as predicted by MOE. (A) Comparison of modeled binding mode of the co-crystallized ligand dihydrotanshinone I (magenta sticks) and its superposed docking conformation (simon sticks). (B) Comparison of modeled binding mode of Kaempferol 3-o-robinobioside (simon sticks) and dihydrotanshinone I (magenta sticks). (C) Detailed binding mode of Kaempferol 3-o-robinobioside (simon sticks) displaying hydrogen bonds (black dashed line) with the key amino acid residues (cyan sticks). (D) 2D depiction of Kaempferol 3-o-robinobioside binding interactions with the key amino acid residues. (E) Estimated free energies of binding of the compounds to acetylcholinesterase binding site.

The computed estimated free energies of binding (S) for the docking of the 3D structures of the eight isolated compounds revealed high affinities towards the active site of acetylcholine esterase. Kaempferol 3-o-robinobioside showed the highest affinity (-8.26 kcal/mol), followed by kaempferol 3-o-gulucoside (-7.64 kcal/mol) (Figure 2).

### 3.3 Pharmacological study

#### 3.3.1 In-vitro assays

##### 3.3.1.1 Antioxidant activity

*U. pumila* extract displayed high free radical scavenging activity with IC_50_ = 7.9 μg/mL while that of ascorbic acid was 5.6 μg/mL (r^2^ = 0.94) in DPPH assay, moderate superoxide anion radical inhibitory activity with IC_50_ = 96.3 ± 2.6 μg/ml while quercetin has IC_50_ = 45.3 ± 1.6 μg/ml and reducing power of 0.42 mM Trolox equivalent antioxidant capacity (TEAC) per mg of *U. pumila* extract.

##### 3.3.1.2 Acetylcholinesterase (ACHE) inhibitory assay

**Figure 3:**
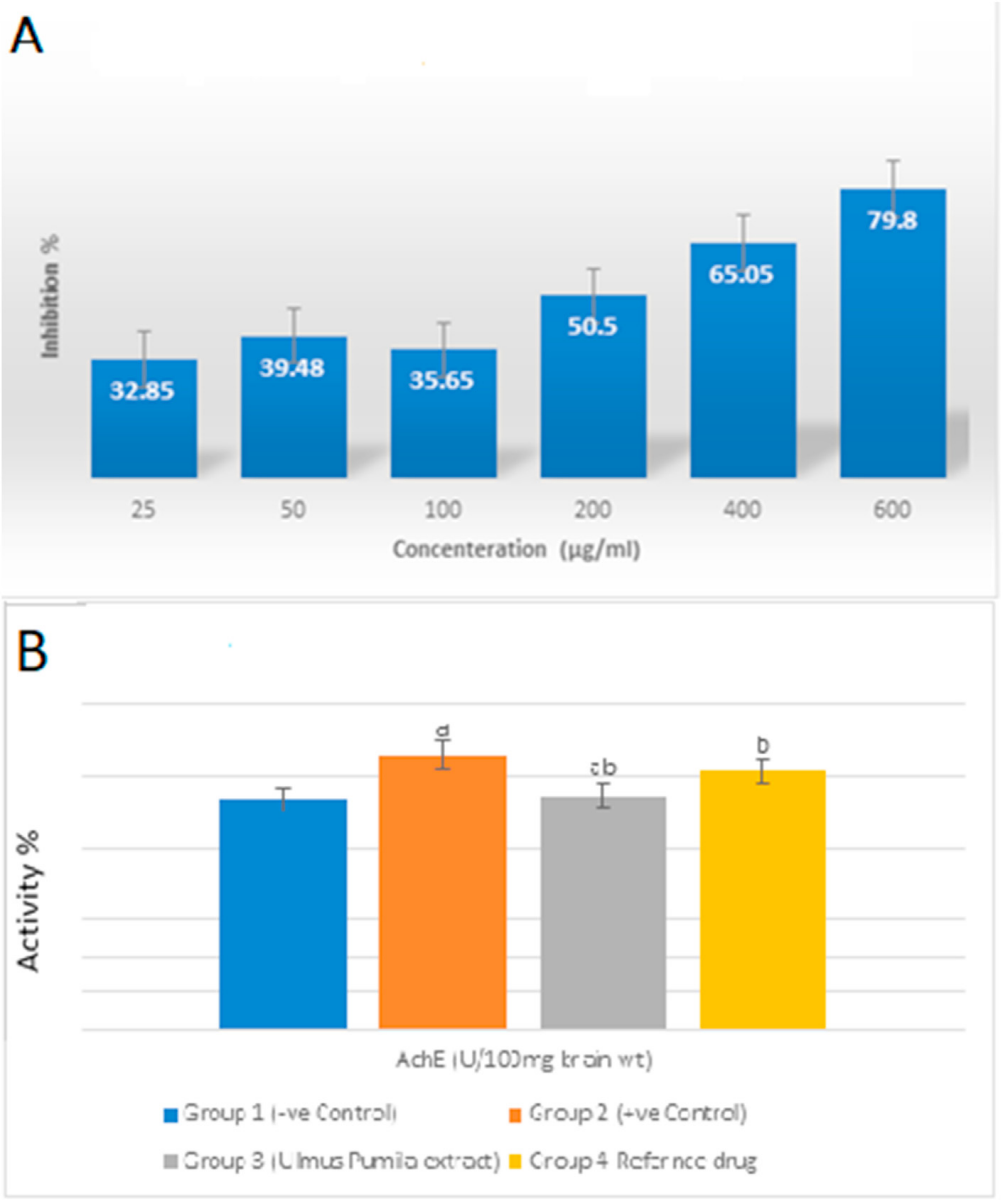
*In-vitro* and *in-vivo* effects of *U. pumila* extract on acetylcholinesterase. A: Effect of *U. pumila* extract on *in-vitro* acetylcholinesterase inhibition. AChE inhibitory activity is expressed as % inhibition. Values are expressed as mean ± SEM, n = 3 for each concentration. B: Effect of *U. pumila* extract on *in-vivo* acetylcholinesterase activity in brain tissue homogenate. Data are represented as Mean ± SEM at n = 6. a Significance change from control group at *p* < 0.05. b Significance change from AD group at *p* < 0.05. c Significance change from Distigmine group at *p* < 0.05.

*U. pumila* extract exerted a dose dependent inhibition of acetyl cholinesterase enzyme *in-vitro* with an IC_50_ of 133.6 ± 0.14 whereas distigmine bromide showed an IC_50_ of 26.83 ± 0.02 (Figure 3A). Compounds 1, 2, 3, 5 and 8 were also investigated for their inhibitory effect on acetyl cholinesterase enzyme and the results were compiled to Table 1. The results showed high inhibition percentages for all investigated compounds however compound (3) showed the maximum effect with IC_50_29.03 ± 0.0155 μM.

**Table 2:** *In-vitro* acetylcholinesterase inhibition percentages for compounds isolated from *U. pumila* extract.

Compound	% Inhibition				
	50μM	25μM	12.5μM	6.25μM	IC50
(+)-Catechin (1)	70.3 ± 1.2	32.5 ± 2.5	16.2 ± 2.4	4.6 ± 1.0	33.61 ± 0.0295
(-) Epicatechin (2)	73.6 ± 1.8	33.7 ± 1.2	19.6 ± 2.6	4.7 ± 0.7	31.62 ± 0.04
Kaempferol-3-o-β-D-glucoside (3)	79.3 ± 0.8	39.4 ± 0.8	14.6 ± 1.2	3.7 ± 0.6	29.03 ± 0.0155
Quercetin-3-O-β-D-glucoside ((isoquercetin) (5)	72.8 ± 2.6	36.3 ± 1.6	14.4 ± 0.9	3.9 ± 1.0	31.72 ± 0.017
Kaempferol 3-o-robinobioside (8)	59.3 ± 2.9	19.9 ± 1.8	4.4 ± 0.9	1.5 ± 0.8	43.13 ± 0.13
Reference drug	75.8 (±1.0)	45.5 (±0.9)	20.8 (±1.5)	10.1 (±0.8	26.83 ± 0.02

*In-vitro* Acetylcholine inhibition activity of the isolated compounds and reference drug; Distigmine Bromide IC_50_: inhibitory concentration 50 and expressed as μM/ml. Values are expressed as mean ± SEM, n = 3 at four different concentration for the tested compounds (100 μg/ml for the reference drug).

#### 3.3.2 Animal study

##### 3.3.2.1 Acute toxicity

The results of the acute lethal toxicity test showed the safety of the oral ingestion of *U. pumila* extract with LD50 of 1500 mg/kg mice body weight. The extract induced no alterations in the general appearance and behavior of mice.

##### 3.3.2.2 Behavioral study

Oral administration of AlCl_3_ dramatically increased the mean time spent by rats in the Y maze to reach the food reward (150% of normal time) suggesting decreased learning and memory functions. However, treatment of animals with *U. pumila* extract resulted in the improvement of memory loss and spatial recognition as expressed by the decrease in the time taken by the animals to reach the food reward (138% from normal time) as compared to AlCl_3_ group.

##### 3.3.2.3 Monoamine neurotransmitters

**Figure 4:**
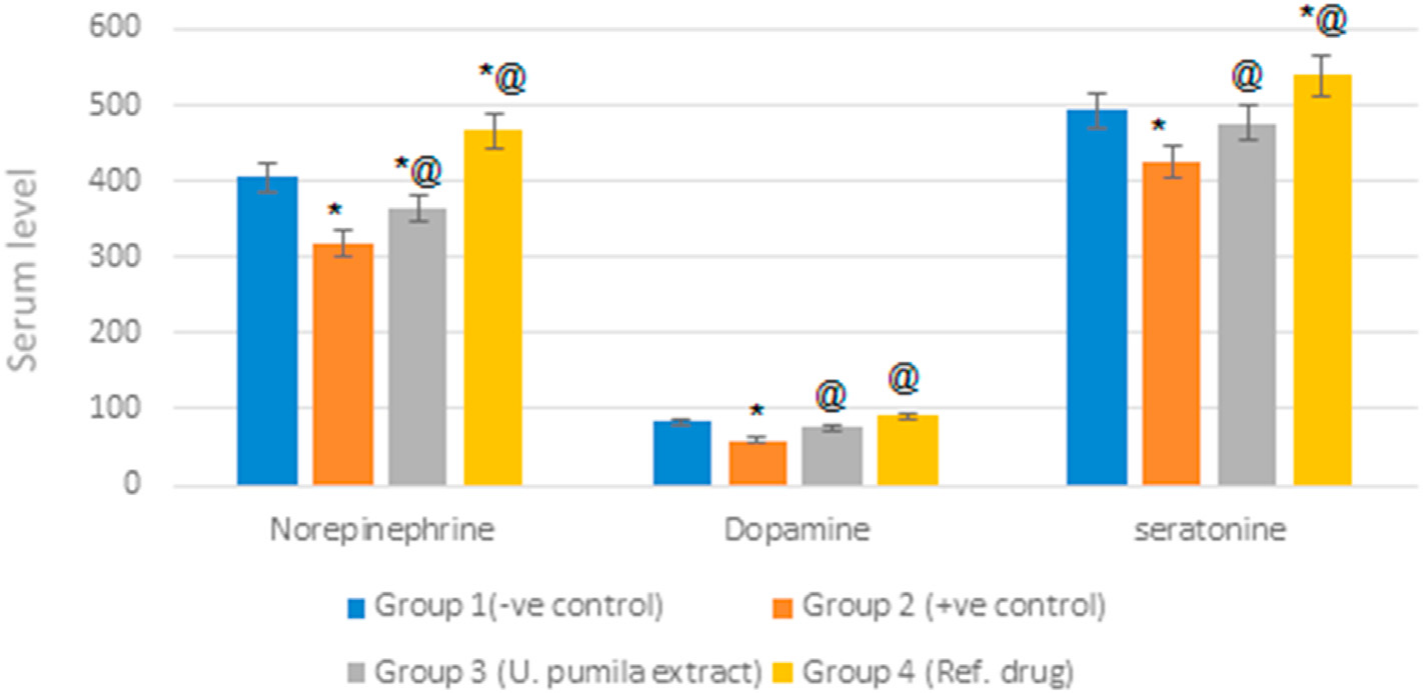
Effect of *U. pumila* extract on monoamine neurotransmitters level in serum of AlCl_3_-induced AD rats. Data are represented as mean ± SEM. Statistical analysis was carried out by one-way analysis of variance (ANOVA) followed by Tukey-Kramer test for multiple comparisons. (n = 6). ∗Significantly different from normal control group at P < 0.5.@ Significantly different from positive control group at P < 0.5.

Induced AD in rats was associated with a marked decrease in monoamine neurotransmitters levels. Norepinephrine, dopamine and serotonin levels decreased by 79%, 71% and 86% as compared to normal rats. Treatment with *U. pumila* alcoholic extract resulted in a significant elevation in the serum levels of neurotransmitters; norepinephrine, dopamine and serotonin by 14%, 29% and 12.5% as compared to the positive control group. Nevertheless, the activity of the reference drug was still higher than *U. pumila* extract (Figure 4).

##### 3.3.2.4 Acetylcholinesterase activity

AlCl_3_ administration resulted in a significant increase in the AChE activity with respect to normal group (119%). Meanwhile, *U. pumila* extract significantly reduced AChE activity reaching to normal value. These results show that administration of *U. pumila* extract suppressed the increase of AChE activity by AlCl_3_ administration (Figure 3B).

##### 3.3.2.5 Antioxidant parameters

AlCl_3_ neurotoxicity is accompanied with dramatic variations in the brain and serum contents of MDA, GSH, CAT and SOD. Whereas AlCl_3_ induced marked elevation in the level of MDA in brain homogenate and serum (4.28 and 2.45 folds, respectively), it induced a significant reduction in the levels of GSH, CAT and SOD by 33%, 73% and 66% in brain tissue. On the other hand, *U. pumila* extract exerted a potent antioxidant effect as demonstrated by the reduction of MDA by 49% as compared to the AlCl_3_ group at significance level *p < 0.05* and the elevations of GSH, CAT and SOD reaching 2.7, 1.5 and 1.6 folds, respectively, when compared to positive control group (AlCl_3_ group) at significance level *p < 0.05* (Table 2).

**Table 1:** Effect *U. pumila* extract on oxidative stress status in brain-homogenate and sera in AlCl_3_-induced AD rats.

GROUPS	BRAIN HOMOGENATE				SERUM		
	MDA (nmol/g. tissue)	GSH (mmol/g. tissue)	CAT (U/g. tissue)	SOD (U/g. tissue)	MDA (nmol/mg protein)	GSH (nmol/mg protein)	CAT (U/mg protein)
NORMAL GROUP	0.5 ± 0.3	0.7 ± 0.01	2.61 ± 0.05	31.88 ± 0.4	0.55 ± 0.023	0.61 ± 0.04	2.34 ± 0.07
AD GROUP	2.14 ± 0.4∗	0.23 ± 0.06 ∗	1.9 ± 0.06∗	21.06 ± 0.5∗	1.35 ± 0.13 ∗	0.32 ± 0.02∗	1.74 ± 0.08 ∗
*U. PUMILA* EXTRACT TREATED GROUP	1.05 ± 0.21^@#^	0.63 ± 0.1^@#^	2.22 ± 0.18 ^@#^	33.24 ± 4.3^@#^	0.97 ± 0.06 ^@#^	0.4 ± 0.05 ^@#^	2.05 ± 0.05 ^@#^
REF. DRUG TREATED GROUP	0.88 ± 0.08^@^	0.71 ± 0.05^@^	2.33 ± 0.47^@^	38.16 ± 1.4^@^	1.12 ± 0.03^@^	0.46 ± 0.021^@^	2.18 ± 0.04^@^

All data are represented as Mean ± SEM at N = 6. ∗ Significance change from control group at *p* < 0.05. @ Significance change from AD group at *p* < 0.05. # Significance change from Distigmine group at *p* < 0.05. (%): Percent of difference from control group.

##### 3.3.2.6 Neurotrophic factors

**Figure 5:**
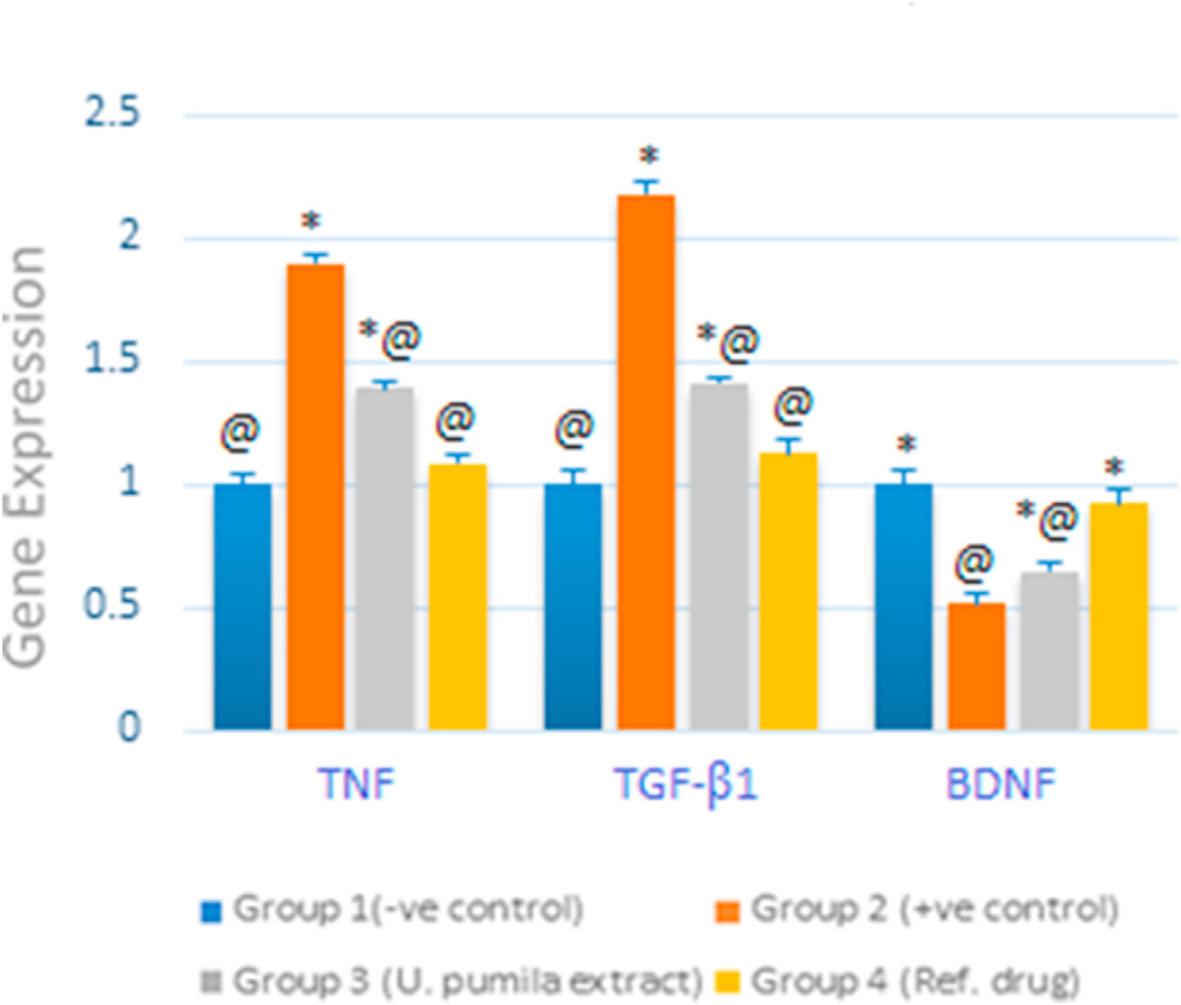
RT-qPCR validation of mRNA expression for BDNF, TGBF-β1 and TNF in brain tissue of *U. pumila* extract treated AlCl_3_-induced AD rats. Data are represented as mean ± SEM. Statistical analysis was carried out by one-way analysis of variance (ANOVA) followed by Tukey-Kramer test for multiple comparisons. (n = 6). ∗^,@^ different letters indicated significantly differences between means at P < 0.05 and error bars represents standard error of mean (SEM).

**Figure 6:**
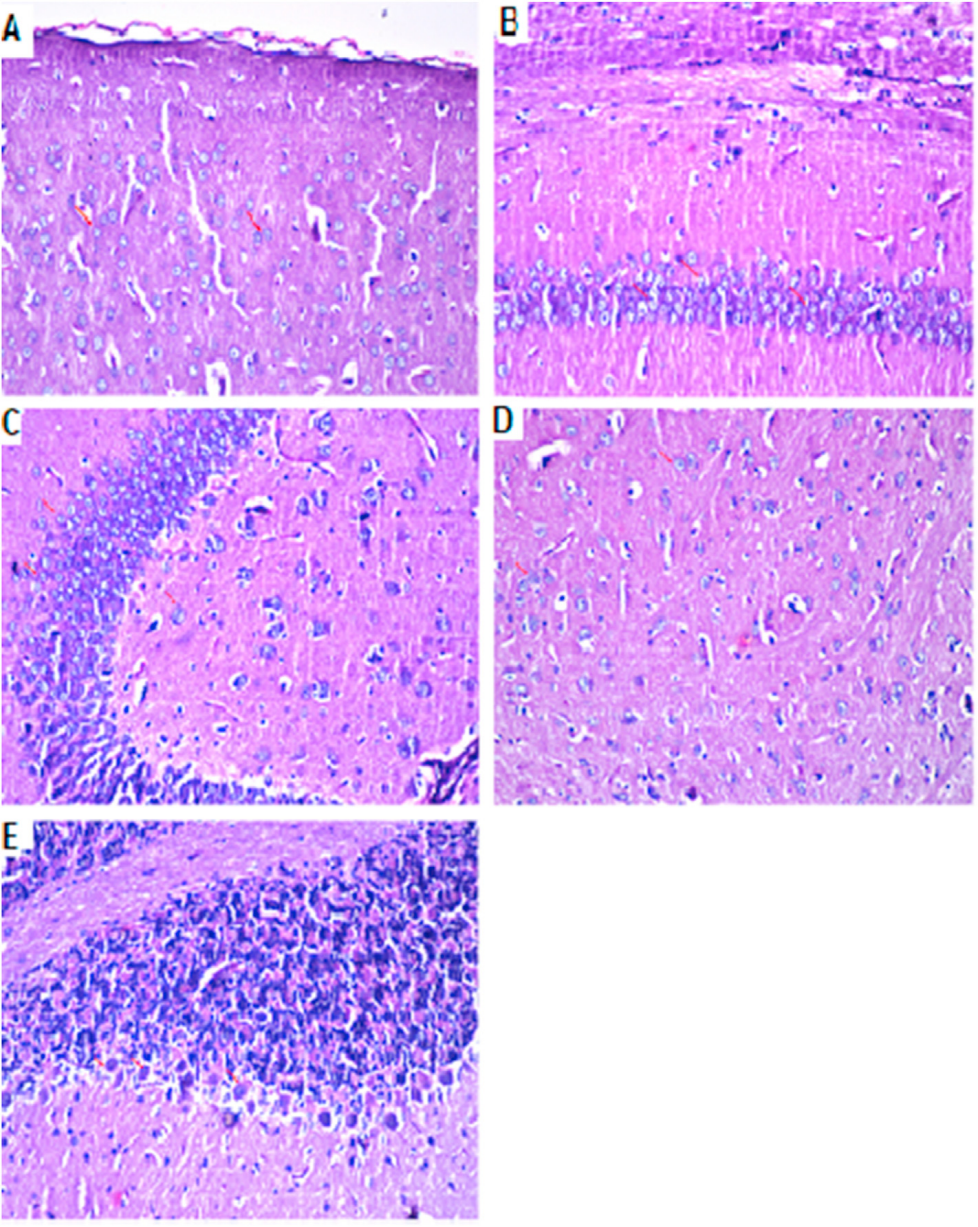
Brain histopathology of group 1: normal control group: cerebral cortex (A), subiculum in hippocampus (B), fascia dentata and hilus in hippocampus (C), striatum (D) and cerebellum (E). (H&E staining, x10, scale bar = 50μm).

The genetic expression of BDNF decreased significantly after the administration of AlCl_3_ in positive control group (51% as compared to normal group) whereas TGF-β1 and TNF expressions were highly elevated; almost duplicated, indicating the establishment of AD in the treated rats. Ingestion of *U. pumila* extract (150 mg/kg b.wt) for six weeks elicited the upregulation of BDNF mRNA expression along with the downregulation of both TGF-β1 and TNF mRNAs expression in a statistically significant manner (P < 0.5) reverting almost to normal values; 92%, 112% and 108% for BDNF, TGF-β1 and TNF, respectively as compared to normal rats (Figure 5) (see Figure 6).

##### 3.3.2.7 Histopathological findings

**Figure 7:**
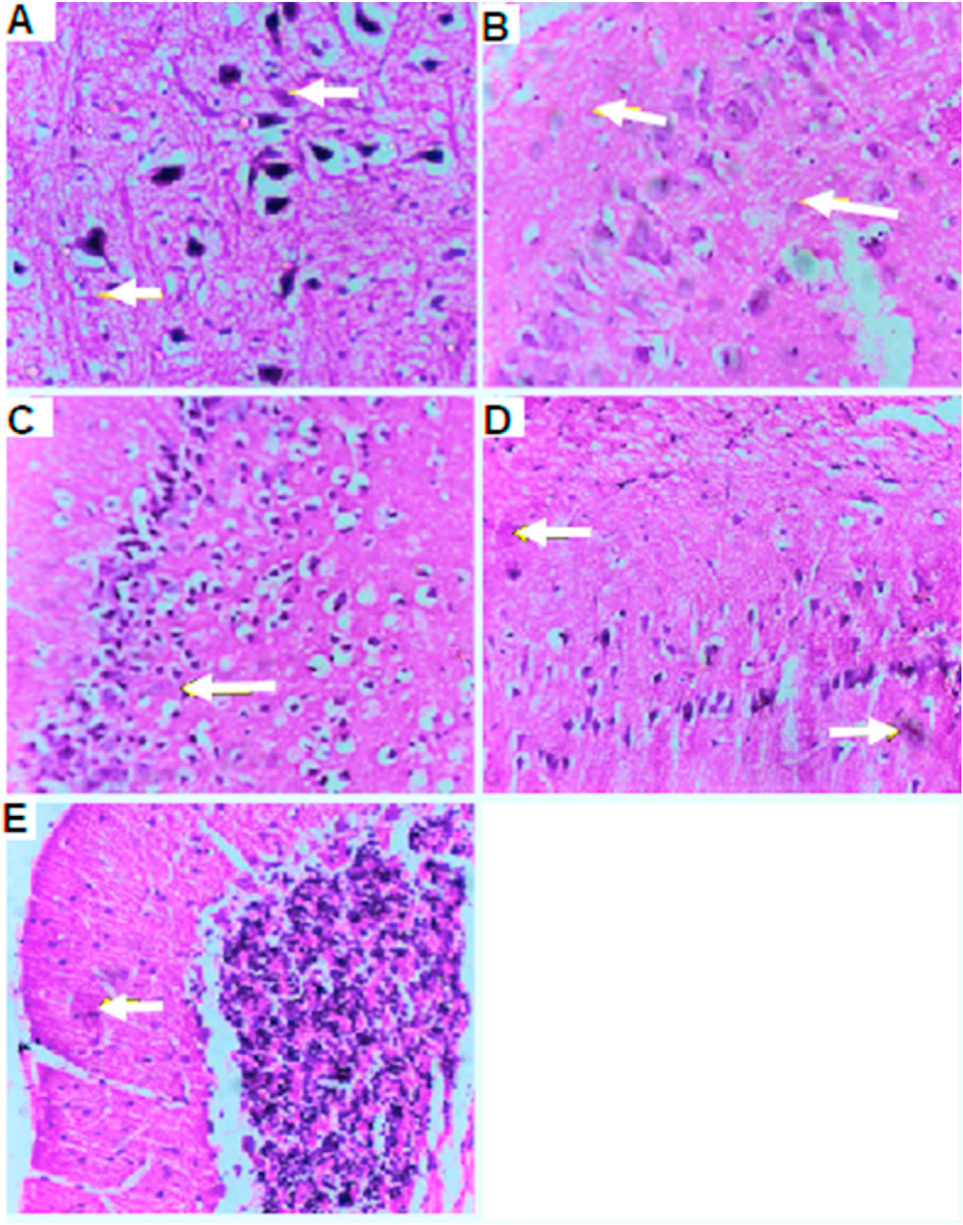
Brain histopathology; group 2: positive control group; rats treated with AlCl_3_ (17 mg/kg b.wt.); cerebral cortex (A), subiculum in hippocampus (B), fascia dentata and hilus in hippocampus (C), striatum (D) and cerebellum (E). (H&E staining, x10, scale bar = 50μm). AlCl_3_-induced extensive neuronal vacuolation and necrosis of the cerebral cortex (indicated by white arrows). The hippocampus showed extensive nuclear pyknosis and degeneration (indicated by white arrows). Striatum displayed multiple focal eosinophilic plagues formation with loss of the neurons.

**Figure 8:**
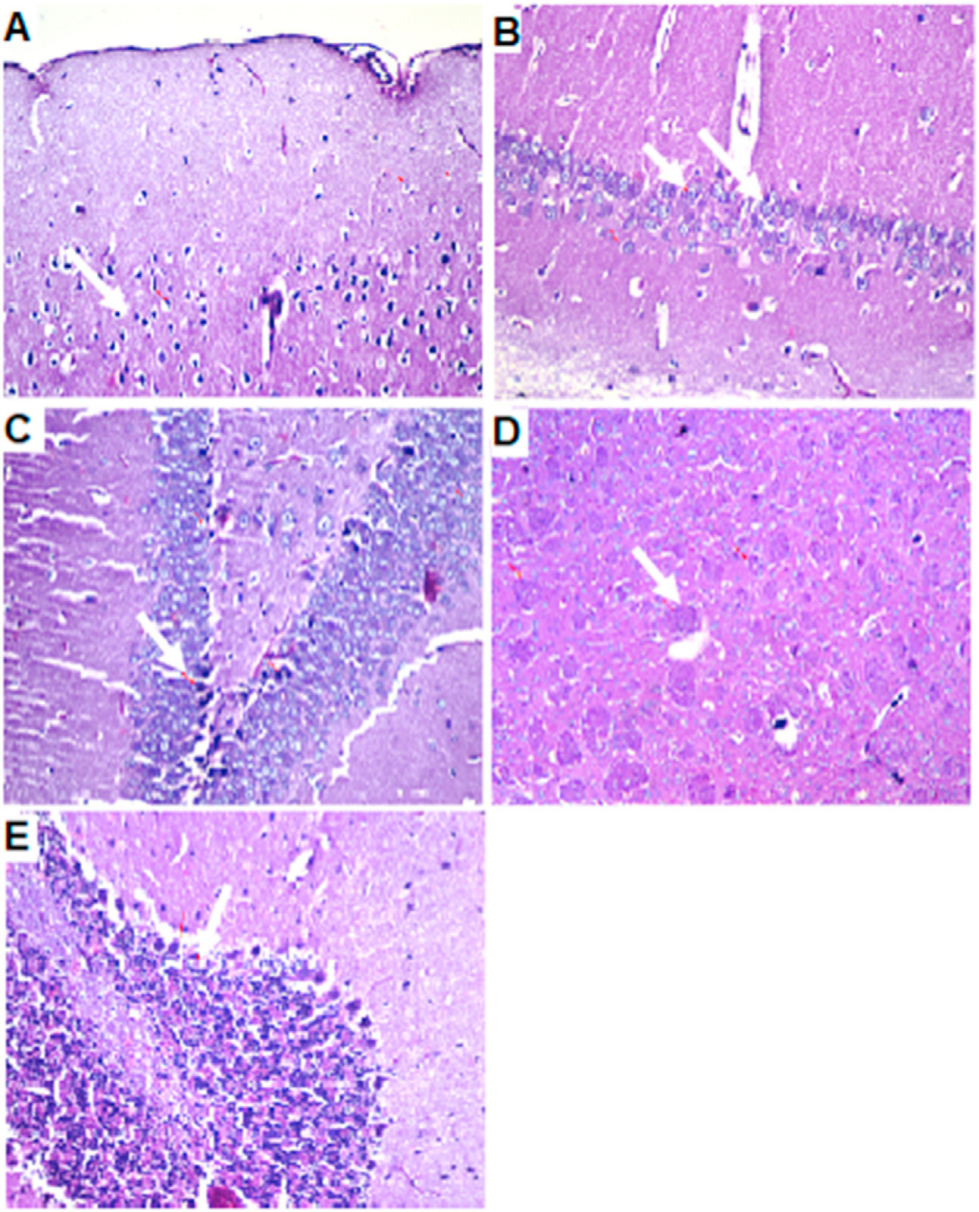
Brain histopathology; Group 3: group of experimentally inducted and treated by *U. pumila* extract (150 mg/kg), cerebral cortex (A), subiculum in hippocampus (B), fascia dentata and hilus in hippocampus (C), striatum (D) and cerebellum (E). (H&E staining, x10, scale bar = 50μm). *U. pumila* extract (150 mg/kg b.wt.) caused limited nuclear pyknosis and degeneration in cerebral cortex with no histopathological alteration in the subiculum in hippocampus. The fascia dentata and hilus in hippocampus showed few neurons with nuclear pyknosis and degeneration. Striatum with multiple focal small eosinophilic plagues formation with loss of the neurons was observed (indicated by white arrows). The cerebellum had no histopathological alteration.

**Figure 9:**
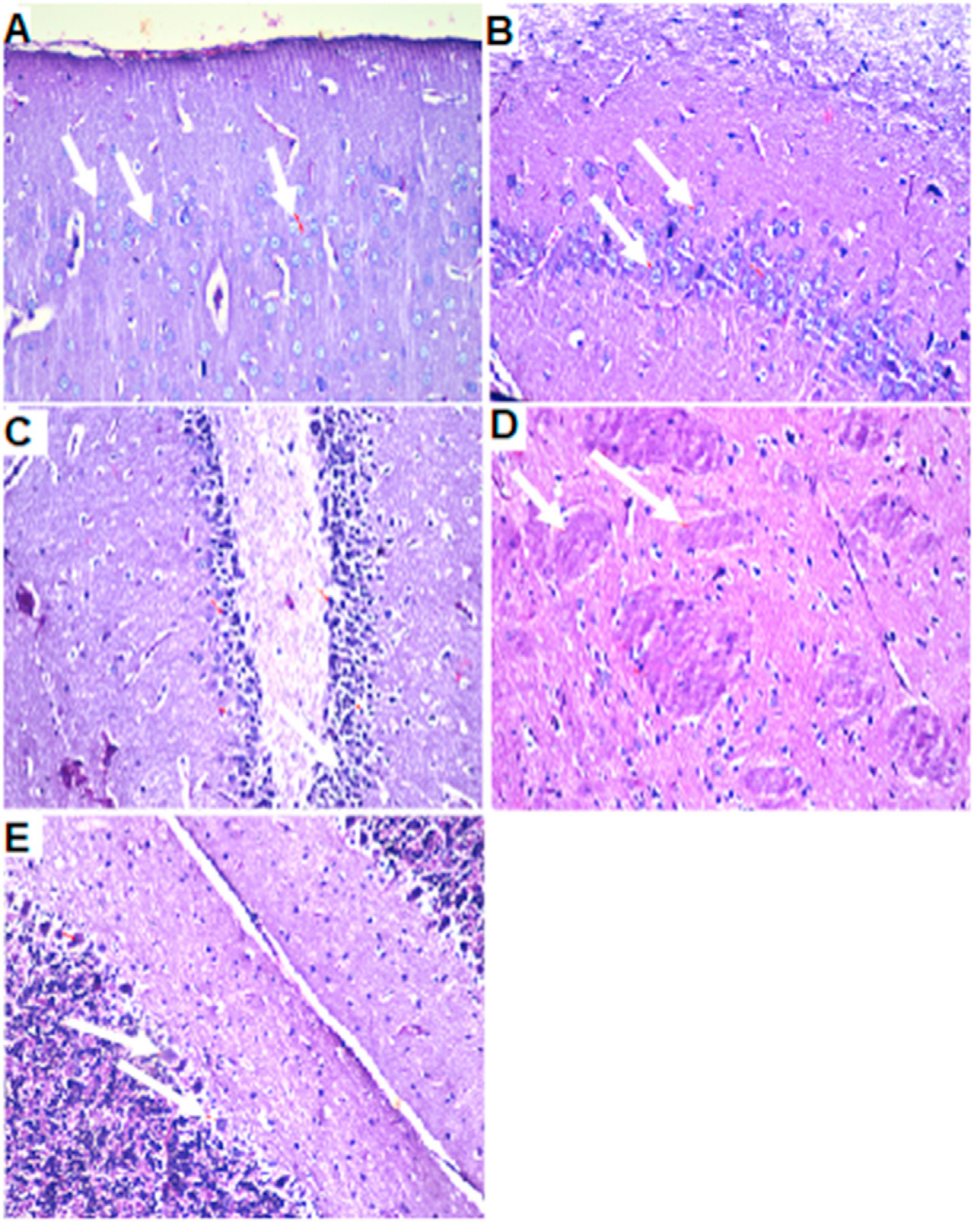
Brain histopathology; Group 4: group of rats experimentally inducted and treated by reference drug: cerebral cortex (A), subiculum in hippocampus (B), fascia dentata and hilus in hippocampus (C), striatum (D) and cerebellum (E). (H&E staining, x10, scale bar = 50μm). Cerebral cortex and Subiculum in hippocampus showed no histopathological alteration. Most neurons of the Fascia dentata and hilus in hippocampus showed nuclear pyknosis and degeneration (indicated by white arrows). The striatum showed multiple focal eosinophilic large plagues formation with loss of the neurons was noticed. The cerebellum recorded no histopathological alteration.

AlCl_3_-induced extensive neuronal vacuolation and necrosis of the cerebral cortex (Figure 7 indicated by white arrows). The hippocampus showed extensive nuclear pyknosis and degeneration (Figure 7B & C- indicated by white arrows). Striatum displayed multiple focal eosinophilic plagues formation with loss of the neurons (Figure 7D). *U. pumila* extract (150 mg/kg b.wt.) caused limited nuclear pyknosis and degeneration in cerebral cortex with no histopathological alteration in the subiculum in hippocampus (Figure 8B). The fascia dentata and hilus in hippocampus showed few neurons with nuclear pyknosis and degeneration (Figure 8C). Striatum with multiple focal small eosinophilic plagues formation with loss of the neurons was observed (Figure 8D- indicated by white arrows). The cerebellum had no histopathological alteration as recorded in (Figure 8E). Cerebral cortex and Subiculum in hippocampus showed no histopathological alteration in the reference drug group as recorded in (Figure 9A, B). Most neurons of the Fascia dentata and hilus in hippocampus showed nuclear pyknosis and degeneration (Figure 9C indicated by white arrows). The striatum showed multiple focal eosinophilic large plagues formation with loss of the neurons was noticed (Figure 9D). The cerebellum recorded no histopathological alteration (Figure 9E).

## 4 Discussion

The current study involves the investigation of the therapeutic potential of the alcoholic extract of *U. pumila* leaves on AlCl_3_-induced AD in rats. The phytochemical analysis of *U. pumila* extract showed the abundance of phenolic compounds. The spectrophotometric estimation of total phenolic and total flavonoid contents revealed remarkably high amounts of phenols; 175.9 ± 5.2mg gallic acid equivalent/gram extract and total flavonoid content of 68.7 ± 1.2 mg rutin equivalent/g extract. The extract was subjected to repeated chromatographic separations resulting in the isolation and purification of eight compounds; catechin, epicatechin, kaempferol-3-o-β-D-glucoside, kaempferol-3-o-β-D-galactoside, quercetin-3-O-β-D-gulucopyranoside, quercetin-3-O-β-D-galactopyranoside, kaempferol-3-o-rutinoside and kaempferol 3-o-robinobioside as identified through spectroscopic analysis.

The predisposing factors for AD are known to comprise a number of genetic aspects. The scientific quest for the identification of these factors led to the identification of genetically related deficit in neurotrophic factors in the AD brain; as brain-derived neurotrophic factor (BDNF) and transforming-growth-factor-β1 (TGF-β1). BDNF plays an important role in the growth, maturation and persistence of neurons. Additionally, BDNF exerts a vital role in synaptic plasticity in brain; the connectivity between neurons through cell-to-cell communication (Bosco et al., 2013). Previous studies demonstrated that induction of AD model leads to decrease in BDNF mRNA levels in the hippocampus as well as impaired bidirectional transport of BDNF in dendrites leading to reduced synaptic efficacy (Prakash and Kumar, 2014) (Gan and Silverman, 2015).

TGF- β1 is one of neurotrophic agents which induces neuroprotective action opposing β-amyloid-induced neurodegeneration where mal signaling of TGF-β1 is believed to be involved in early stages in the pathogenesis of AD (Bosco et al., 2013). Some discrepancy was found in literature concerning the expression of TGF-β1 in AD Whereas, Chen et al. (2015) reported that TGF-β1 level in the plasma of AD patients is reduced Wyss-Coray et al. (2000), reported the chronic excessive production of TGF-β1 in AD transgenic mice (Chen et al., 2015) (Wyss-Coray et al., 2000). Recent findings have shown abnormal increase of the levels of TGF-β1 in the brain of patients suffering AD, associated with neuroinflammation, accumulation of extracellular matrix compounds and cerebrovascular stiffness, neuronal apoptosis along with the development of vascular hypertrophy (Ongali et al., 2010). In the present work, a marked decrease has been noticed in the level of BDNF mRNA in the group of animals receiving AlCl_3_. Oral treatment of the AD rats with *U. pumila* extract group reverted this neurotrophic factor to normal levels as it showed a significant increase of BDNF mRNA comparable to the normal control group. A marked decrease was also noticed in the gene expression of TGF- β1 in *U. pumila* treatment group reverting to normal levels after the prominent elevation due to AlC_3_ intoxication.

Additionally, recent research drew the attention to cytokine-mediated neuroinflammation in AD, as tumor necrosis factor α (TNF-α). Although the levels of TNF-α in the periphery and central nervous system of healthy adults are maintained at very low levels, the levels of this cytokine are significantly elevated in blood and brain of patients with AD, and many clinical and animal studies have demonstrated a link between excess TNF-α levels in the brain and AD (Chang et al., 2017). In the current study TNF mRNA was over expressed in AlCl_3_ treated rats which affirms previous literature. Our results showed that *U. pumila* extract reduced the expression of TNF suggesting the suppression of the inflammatory cascade.

*U. pumila* extract alleviated cognitive status as deduced from behavioral profile in Y maze. Y-maze analysis has been shown to be a reliable, noninvasive test to determine cognitive changes in Wistar rats through the measurement of the spontaneous alternation behavior in the Y-maze task (Hritcu et al., 2012). Results revealed that AlCl_3_- intoxicated rats displayed dementia and retarded learning ability whereas the administration of *U. pumila* extract resulted in the improvement of memory loss and spatial recognition as expressed by latency time.

Cognitive processes such as concentration and learning have been related to biogenic amine neurotransmitters; norepinephrine, dopamine and serotonin. Several studies have shown that levels of brain neurotransmitters have decreased in AD. Administration of AlCl_3_ impaired multiple neurotransmitter system *viz.* serotonergic and dopaminergic system (Foyet et al., 2015). The present study showed that oral treatment of rats with *U. pumila* extract (150 mg/kg) for six weeks exhibited an increase in the serum levels of norepinephrine, dopamine and serotonin.

On the other hand, the elevated activity of AChE leads to increased degradation of acetylcholine (Ach) neurotransmitter which in turns declines the ACh pool in the brain which is essential in learning and memory. AlCl_3_ administration amplifies the AChE activity which is one of the major causes for the cholinergic deficit occurrence after its administration (Dong et al., 2007). In this study we found that treatment with *U. pumila* extract significantly reduced the AChE activity in rats' brains as compared to the AlCl_3_ treated animals. It reveals that inhibition of AChE activity by the plant extract had a protective role in acetylcholine degradation and improved the cholinergic neurotransmission. Additionally, *U. pumila* extract and the major isolated compounds; kaempferol 3-o-β-D-glucoside, kaempferol 3-o-robinobioside, quercetin-3-O-β-D-gulucopyranoside, catechin and epicatechin, exerted a significant acetylcholinesterase inhibitory action in *in-vitro* assay. The virtual molecular docking of the 3D structures of the compounds on AChE active sites showed that kaempferol 3-o-robinobioside had the highest affinity towards the receptor, nevertheless, kaempferol 3-o-β-D-glucoside showed the highest inhibitory effect of AChE *in-vitro* with IC_50_ of 29.03 ± 0.0155.

Thus, the plant extract reduced the cholinergic deficits produced by AlCl_3_ administration resulting in an enhanced neuroprotective effect. Inhibition of acetylcholinesterase (AChE) is currently the conformist strategy for the treatment of AD, senile dementia, ataxia, and Parkinson's disease.

Multiple reports proved that brain tissues in AD patients endures high levels of oxidative stress throughout the progression of the disease. Oxidative damage in the brain leads eventually to the development of Alzheimer's disease through oxidation of proteins, lipids and DNA (Feng and Wang, 2012). It is reported that aluminum toxicity occurs through the potentiation of activity of Fe^2+^ and Fe^3+^ ions causing oxidative damage. Aluminum further induces the activation of a cascade of redox-sensitive cell signal pathways. Brain cells are known to contain a very high percentage of long chain polyunsaturated fatty acids which are regularly subject to free radical-induced lipid peroxidation leading to the accumulation of reactive oxygen species (ROS) (Foyet et al., 2015).

*U. pumila* extract exerted high antioxidant capacity *in-vitro*; DPPH free radical scavenging assay (IC_50_ = 7.9 μg/mL), superoxide anion radical scavenging activity (IC50 96.3 ± 2.6 μg/ml) and reducing power assay (0.42 mM TE/mg). The oral administration of AlCl_3_ (17 mg/kg, p.o.) for four weeks resulted in elevated oxidative stress. The results of antioxidant study showed elevated brain MDA and decreased brain levels of SOD, CAT, and GSH in the AlCl_3_-induced AD rats compared to normal rats. After six weeks treatment with *U. pumila* extract (150 mg/kg), there was a significant improvement of the levels of SOD, CAT and GSH whereas MDA level was decreased significantly. These results suggest that the plant extract had an *in vivo* antioxidant activity and is capable of ameliorating the effect of reactive oxygen species (ROS) in the brain of rats.

The oral administration of AlCl_3_ (17 mg/kg, p.o.) for four weeks resulted in extensive neuronal vacuolation and necrosis of the cerebral cortex, extensive nuclear pyknosis and degeneration of the hippocampus and the formation of multiple focal eosinophilic plagues in the striatum. The histopathological examination of the brain tissues from different sections revealed improvement in the cerebral cortex, the hippocampus and striatum of the *U. pumila* treated group with minor nuclear pyknosis and less plagues compared to the reference drug; Donepezil*.*

## 5 Conclusion

In sight of the aforementioned findings, *U. pumila* alcoholic extract, and its major flavonoids particularly kaempferol-3-o-β-D-glucoside present new therapeutic candidates for neurodegenerative disorders specifically AD. *U. pumila* alcoholic extract exerts its action through the modulation of neurotrophic factors, cholinergic and monoamine neurotransmission as well as through antioxidant and anti-inflammatory pathways.

## Declarations

### Author contribution statement

R. Hussein: Conceived and designed the experiments; Performed the experiments; Contributed reagents, materials, analysis tools or data; Wrote the paper.

A.H. Afifi: Conceived and designed the experiments; Performed the experiments; Analyzed and interpreted the data; Contributed reagents, materials, analysis tools or data; Wrote the paper.

A.A.F. Soliman and Z.A. El Shahid: Performed the experiments; Analyzed and interpreted the data; Contributed reagents, materials, analysis tools or data.

K.M.A. Zoheir: Performed the experiments; Contributed reagents, materials, analysis tools or data.

K.M. Mahmoud: Conceived and designed the experiments; Performed the experiments; Contributed reagents, materials, analysis tools or data; Wrote the paper.

### Funding statement

This research did not receive any specific grant from funding agencies in the public, commercial, or not-for-profit sectors.

### Declaration of interests statement

The authors declare no conflict of interest.

### Additional information

No additional information is available for this paper.
